# Role of Multi-Detector Computed Tomography Indices in Predicting Extracorporeal Shockwave Lithotripsy Outcome in Patients With Nephrolithiasis

**DOI:** 10.7759/cureus.22745

**Published:** 2022-03-01

**Authors:** Aashish Singh, Anil K Sakalecha

**Affiliations:** 1 Radiodiagnosis, Sri Devaraj Urs Educational Trust (SDUAHER), Kolar, IND

**Keywords:** hounsfield density, skin-stone distance, hounsfield units, extracorporeal shockwave lithotripsy, nephrolithiasis, non-contrast computed tomography

## Abstract

Background

Nephrolithiasis is one of the most common renal pathologies and is routinely encountered in daily practice. Non-contrast computed tomography (NCCT) is the gold standard diagnostic imaging modality for urolithiasis. The role of HU (Hounsfield units) in calculus as a predictor of extracorporeal shock wave lithotripsy (ESWL) has been studied in the past. This study aims to evaluate the role of HU value and various other NCCT indices in predicting the outcome of ESWL.

Material and methods

This was a prospective observational study that included 45 patients suffering from nephrolithiasis who underwent NCCT-KUB (kidney, ureter, and bladder) followed by ESWL. The NCCT indices were evaluated and correlated with the outcome of ESWL. NCCT-KUB was performed using multidetector SIEMENS® SOMATOM EMOTION 16-slice CT scanner (SIEMENS, Munich, Germany).

Results

In our study, the HU value turned out to be a statistically significant predictor of ESWL success (p <0.05), and the renal pelvis also proved to be a good prognostic indicator for ESWL success. The cut-off value of <1179 HU favored a successful outcome of ESWL, while if >1179 HU, ESWL is likely to fail. Hence, the successful outcome of ESWL is inversely proportional to the HU value. Renal pelvic calculi (n=14) showed a 100% success rate, which was better than all other calculus locations (p<0.05). However, the rest of the indices did not show any statistical significance.

Conclusion

Multi-detector NCCT-KUB indices can help in the selection of patients with a good prognosis for ESWL, which will prevent the patient from undergoing undesired invasive procedures.

## Introduction

Nephrolithiasis is a commonly encountered renal pathology in routine practice [1]. It poses a significant health burden to the population as it greatly impacts the quality of life [2]. Nearly about 7-10% of the population suffers from nephrolithiasis at least once in their lifetime [3,4]. In India, the incidence of nephrolithiasis is approximately 12% [3,5]. Also, there has been a rise in the occurrence of renal calculi among the pediatric age group in the past few decades [6].

Calculi usually develop in the kidneys but can be encountered at any site within the entire urinary tract. Calcium, uric acid, struvite, and cystine are the common types of calculi, with calcium oxalate/phosphate being the most common [3,7,8]. The typical clinical presentation is that of flank pain, which can radiate to the groin. This pain is usually severe and colicky [7]. Imaging evaluation of nephrolithiasis includes plain radiography, ultrasound, and computed tomography. Non-contrast computed tomography (NCCT) is the gold standard diagnostic imaging modality for nephrolithiasis, which yields additional information or findings such as hydroureteronephrosis, perinephric collection, fat stranding, and other causes of pain [9,10].

The commonly employed treatment options for nephrolithiasis include extracorporeal shock wave lithotripsy (ESWL), retrograde ureteroscopic calculus fragmentation, and percutaneous nephrolithotomy (PCNL). In the year 1980, Chaussy introduced the concept of ESWL to the world [11]. Presently, ESWL is considered the treatment of choice for renal calculi <2 cm; however, the success rates vary from 46% to 91%, which is influenced by numerous factors [12,13].

In India, most of the studies have evaluated only the HU value for prognosticating ESWL outcomes. Other calculus properties, such as calculus size, location, and skin-to-stone distance (SSD), have not been studied in detail in the Indian population. In our study, the aim was to study various CT parameters, their influence on the outcome of ESWL, and the derivation of cut-off values for the same.

## Materials and methods

Source of data

We conducted a prospective observational study on 45 patients with clinically/sonographically suspected nephrolithiasis who were referred for CT evaluation and underwent ESWL at the Department of Radiodiagnosis, R. L. Jalappa Hospital and Research Centre, Kolar. Prior informed consent was obtained from the patients for their willingness to participate in the study. Patients with an age ≥18 years with a calculus size ranging from 5-20 mm were included in the study. Patients with gravid status, coagulopathy, and severe untreated hypertension were excluded from the study. The study was approved by the Institutional Ethics Committee of Sri Devaraj Urs Medical College, Tamaka, Kolar (DMC/KLR/IEC/685 2020-21).

Method of collection of data

The baseline data of the patients participating in the study were recorded. NCCT-KUB (kidney, ureter, and bladder) was performed with SIEMENS® SOMATOM EMOTION 16 slice CT scanner (SIEMENS, Munich, Germany). After performing CT, various parameters such as HU (Hounsfield units), calculus size and location, HD (Hounsfield density), and skin-stone distance (SSD) were determined.

NCCT-KUB indices

**Figure 1:**
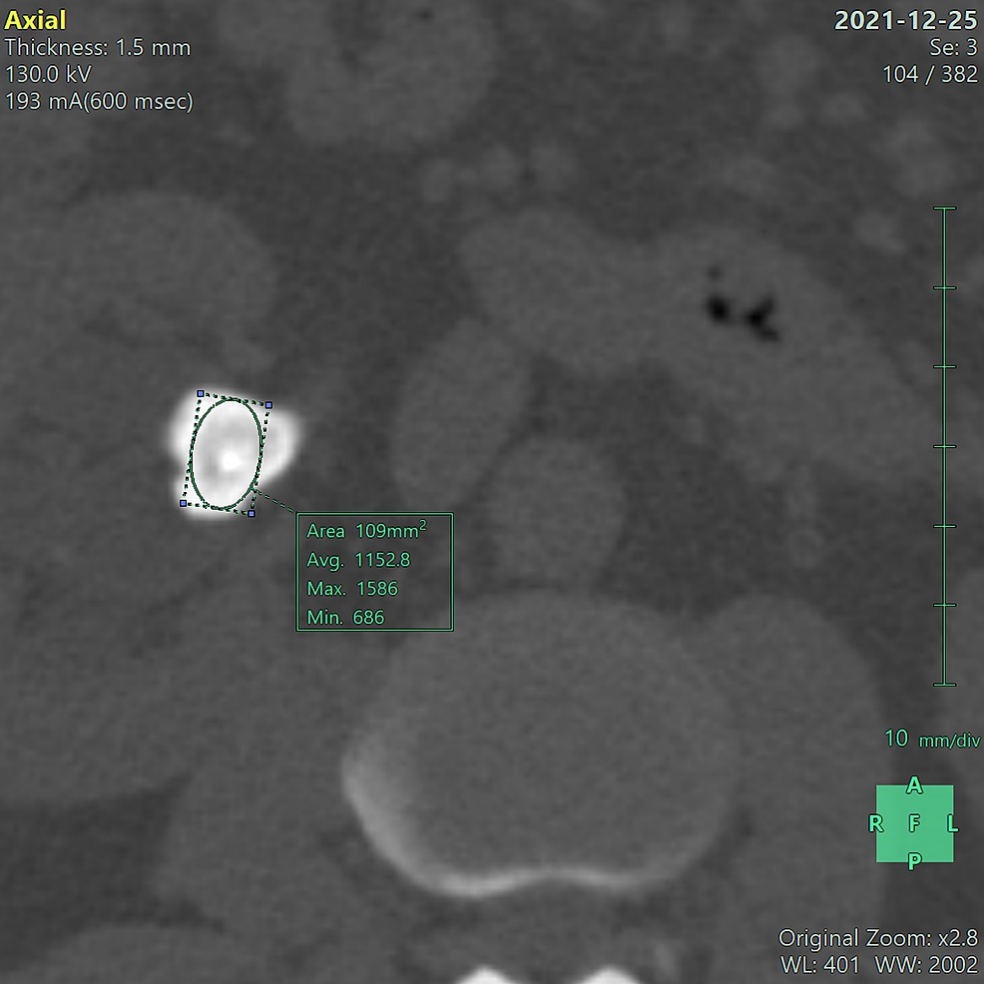
NCCT-KUB (bone window) in axial section shows a calculus in renal pelvis of right kidney with a mean HU value of ~1153.

HU was measured by drawing a region of interest (ROI) in the bone window, covering most of the calculus that displays the minimum, mean, and maximum HU value. The mean/average HU value was considered (Figure 1).

**Figure 2:**
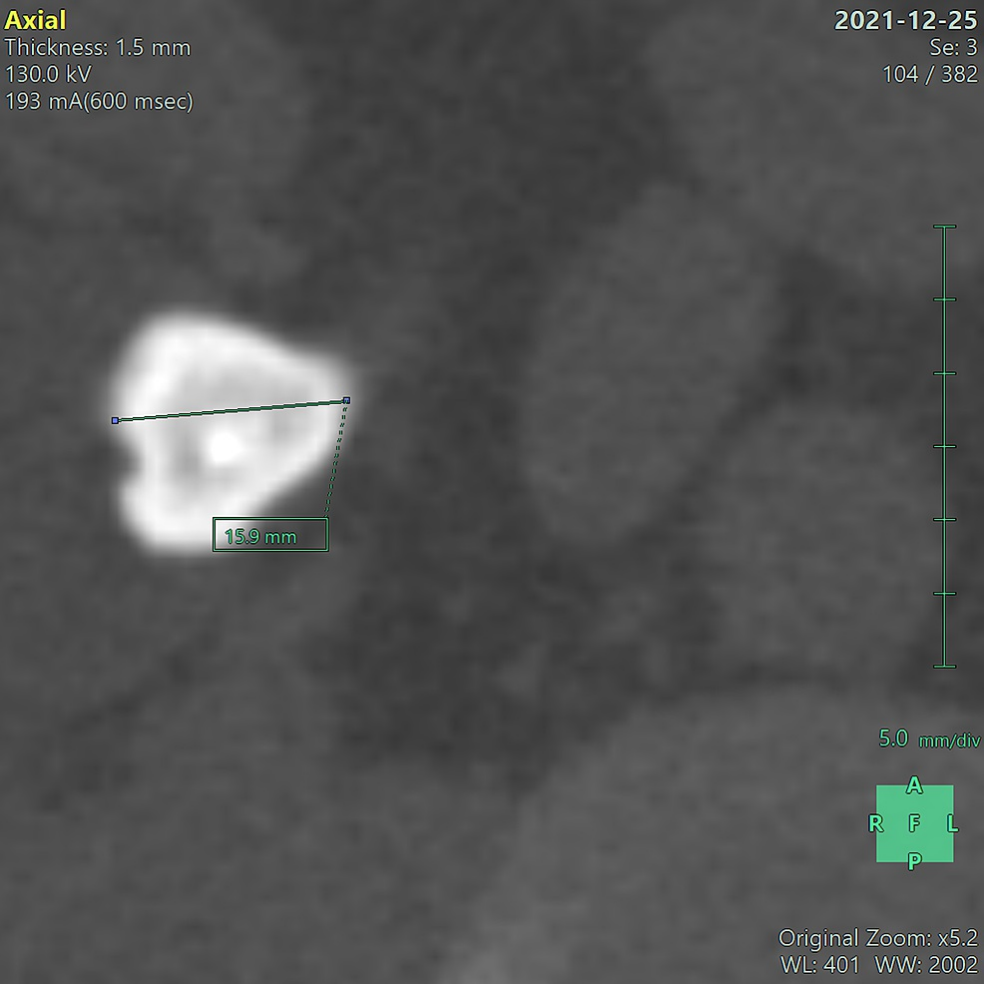
NCCT-KUB shows a calculus size of 15.9 mm. Same patient as in Figure 1.

Calculi were evaluated in all three planes, and the largest dimension was taken as the calculus size. Calculus size was measured at 5× zoom in the bone window setting (Figure 2).

**Figure 3:**
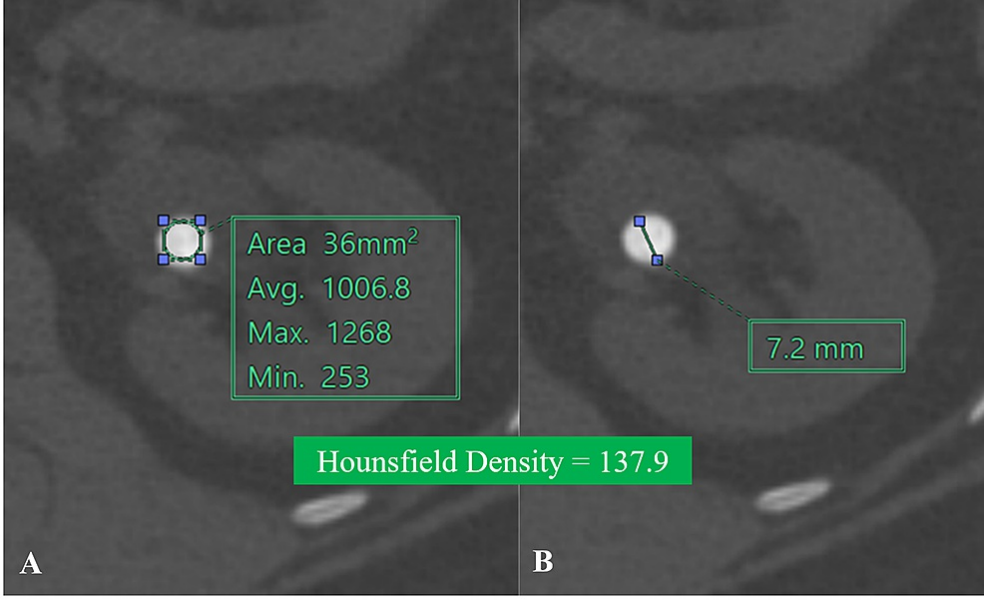
NCCT-KUB (bone window, axial section) shows calculus of 1006.8 HU (A) and 7.2 mm size (B). The calculated Hounsfield density was 137.9 HU/mm.

Based on the calyx in which the calculus is situated, renal calculi locations were classified as upper, inter, and lower poles, and renal pelvis. HD was calculated as the ratio of the HU value of calculus to the largest dimension of calculus. It was expressed as HU/mm (Figure 3).

**Figure 4:**
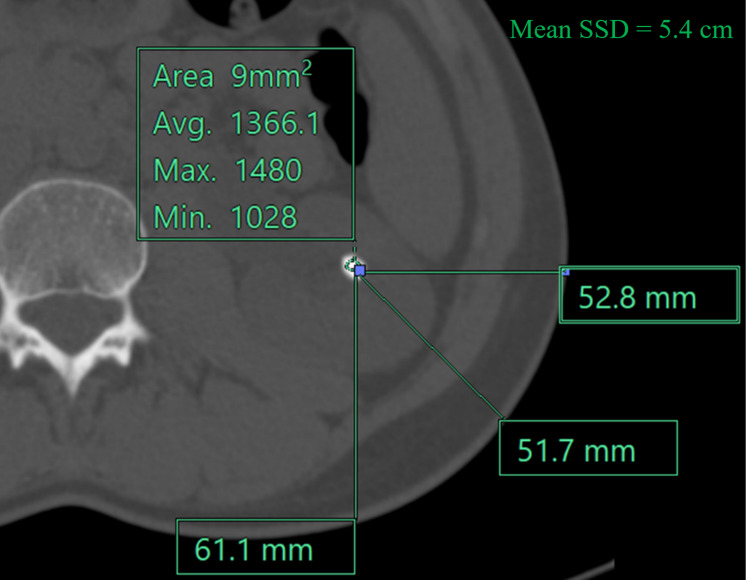
NCCT-KUB (bone window, axial section) showing SSD. Three lines are drawn from the skin surface - horizontal (5.2 cm), vertical - 6.1 cm, and line making 45° angle with both - 5.1 cm. Mean SSD is 5.4 cm.

The SSD is self-explanatory and depends on factors such as body mass index (BMI), abdominal circumference, and the location of the calculus. To determine this parameter, three lines were drawn from the skin surface to the calculus; horizontal, vertical, and a third line, making a 45° angle with both earlier mentioned lines. SSD was taken as the mean of these three distances (Figure 4).

Lithotripter

Extracorporeal shockwave lithotripsy was performed using the SIEMENS SIGMA® ORILITHO-CAL lithotripter. It is equipped with a C-arm and an ultrasound (SIEMENS ACUSON X300). Patients underwent ESWL for a maximum of three sessions. The parameters of the lithotripter were set at 60 shockwaves/minute for 45 minutes at an energy of 20 kV. A follow-up ultrasound was performed to check for absent/fragmented calculus ≤4 mm (which was considered a successful treatment).

Statistical methods

IBM SPSS was used for statistical analysis. The result (success v/s failure) was considered the primary outcome variable. Hounsfield units, Hounsfield density, calculus size and location, and skin-to-skin distance were all considered as secondary outcome variables. Descriptive analysis was carried out by mean and standard deviation for quantitative variables and frequency and proportion for categorical variables. Cross tabulation was performed to determine the relationship between categorical outcome parameters using the Chi-square test. A P-value of <0.05 was considered statistically significant.

## Results

Forty-five patients were analyzed, aging between 18 and 85 years, with a mean age of 41.62 years ± 15.25. Among the 45 patients, 24 were males and 21 were females. Twenty-six patients had calculus in the right kidney, while 19 had calculus in the left kidney.

HU value

The mean Hounsfield units in the study population were 824.36 ± 315.47 HU, ranging from 274 HU to 1338 HU. Out of 42 patients with a successful ESWL outcome, the HU value for 41 (97.62%) of them was <1179.50, and the remaining one (2.38%) had a HU of ≥1179.50. All three ESWL failure cases had an HU of ≥1179.50. As shown in Table 1, a statistically significant difference was observed in Hounsfield units between the results (p-value <0.05).

**Table 1:** Comparison of Hounsfield units between study group (n=45).

Hounsfield units	Result		p-value
	Success (n=42)	Failure (n=3)	
<1179.50	41 (97.62%)	0 (0%)	<0.05
≥1179.50	1 (2.38%)	3 (100%)	

Calculus location

The most common calculus location in our study was renal pelvis (n=14, 31.1%) followed by upper pole (n=12, 26.6%), lower pole (n=12, 26.6%), and interpole (n=7, 15.5%) as shown in Table 2. ESWL showed a 100% success rate for renal pelvic calculi, while there was one case of failure each in the upper, inter, and lower poles. Therefore, the renal pelvis is a favorable location for a successful ESWL outcome (p<0.05).

**Table 2:** Descriptive analysis of calculus location in the study population (n=45).

Location	Frequency	Percentages
Pelvis	14	31.11%
Upper pole	12	26.67%
Lower pole	12	26.67%
Interpole	7	15.56%

Calculus size

The mean calculus size in the study population was 11.46 ± 4.14 mm, ranging from 5.2 mm to 18.8 mm. Out of 42 patients with a successful ESWL outcome, 28 (66.67%) of them had a calculus size of <13.30 mm and the other 14 (33.33%) had a calculus size of ≥13.30 mm. Out of three patients with ESWL failure, one of them had a calculus size of <13.30 mm (33.33%) and the other two had a size of ≥13.30 mm (66.67%). As shown in Table 3, no statistically significant difference was observed in calculus size between the results (p-value >0.05).

**Table 3:** Comparison of calculus size between study group (n=45).

Calculus size	Result		p-value
	Success (n=42)	Failure (n=3)	
<13.30 mm	28 (66.67%)	1 (33.33%)	0.244
≥13.30 mm	14 (33.33%)	2 (66.67%)	

Skin-to-stone distance

The mean skin-to-stone distance in the study population was 8.63 ± 2.97 cm, ranging from 4.5 cm to 24.8 cm. Out of 42 patients with a successful ESWL outcome, 27 of them had a SSD of ≥7.55 cm (64.29%) and 15 had an SSD of <7.55 cm (35.71%). Out of three patients with ESWL failure, one had an SSD ≥7.55 cm (33.33%), and two had an SSD of <7.55 cm (66.67%). As shown in Table 4, no statistically significant difference was observed in the SSD between the results (p-value >0.05).

**Table 4:** Comparison of skin-to-stone distance between study group (n=45).

Skin-to-stone distance	Result		p-value
	Success (N=42)	Failure (N=3)	
≥7.55 cm	27 (64.29%)	1 (33.33%)	0.285
<7.55 cm	15 (35.71%)	2 (66.67%)	

Hounsfield density

The mean Hounsfield density in the study population was 81.04 ± 38.65 HU/mm, ranging from 11.6 HU/mm to 167.3 HU/mm. Out of 42 patients with successful ESWL results, 27 (64.29%) of them had an HD of <89.65, and 15 (35.71%) had an HD of ≥89.65. Out of three patients with ESWL failure, one (33.33%) had an HD <89.65 and two (66.67%) had an HD of ≥89.65. As shown in Table 5, no statistically significant difference was observed in Hounsfield density between the results (p-value >0.05).

**Table 5:** Comparison of Hounsfield density between study group (n=45).

Hounsfield density	Result		p-value
	Success (n=42)	Failure (n=3)	
<89.65	27 (64.29%)	1 (33.33%)	0.285
≥89.65	15 (35.71%)	2 (66.67%)	

HU value turned out to be a statistically significant predictor of ESWL success (p<0.05), while renal pelvic calculi also favored a successful ESWL outcome. HU of 1179.50 and below had a sensitivity of 97.62% and a specificity of 100%. The cut-off value of <1179 HU favored a successful outcome of ESWL, while >1179 HU, ESWL is likely to fail. Hence, the successful outcome of ESWL is inversely proportional to the HU value. Renal pelvic calculi (n=14) showed a 100% success rate, which was better than all other locations. However, other indices did not prove to be statistically significant in predicting the ESWL outcome.

## Discussion

Nephrolithiasis causes significant morbidity and health burden. It has a significant influence on the quality of life [4]. ESWL is the first choice for renal stones <2 cm in size. However, there is variation in the results of different studies. The results of ESWL can be optimized by using certain principles and a proper selection of cases [14]. NCCT-KUB has been revolutionary for the evaluation and management of nephrolithiasis [9].

After its introduction by Chaussy et al. in 1980, ESWL has become extremely renowned in the treatment of nephrolithiasis and is also the treatment modality of choice for renal calculi <20 mm as it is non-invasive in nature [15]. Previous studies have reported varying percentages of ESWL success rates ranging from 46% to 91% (in our study, the overall success rate was 93%) [16].

In our study, we found that out of all the indices, only the HU value emerged as a statistically significant index for prognosticating the outcome of ESWL. Also, calculi in the renal pelvis have a favorable outcome, as ESWL had a 100% success rate in these cases. In a similar study conducted by Elawady et al. in 2021, they concluded that calculi with HU <975 have a better outcome of ESWL [17]. In the present study, the cut-off value HU <1179 was found to favor a successful outcome of ESWL.

Elawady et al. came to the conclusion that calculi in the calyces have a poor ESWL outcome in comparison to renal pelvic and ureteral stones. Also, lower pole calculi had poorer outcomes when compared to middle and upper pole calculi [18]. We observed that the outcomes are better in case of renal pelvis as compared to upper, middle, and lower calyces.

Weld et al. conducted a similar trial to predict ESWL outcome based on patient and stone computed tomographic characteristics and stated that smaller calculi with lower mean HU levels were more successfully fragmented [19]. However, in our study, calculus size was not statistically significant in predicting ESWL success.

Hounsfield density (HD) is one of the less explored parameters in predicting the ESWL outcome. In the current study, HD did not have any significant impact on the results, as successful cases had a wide range of HD values. Magnuson et al. studied the correlation between ESWL success and HD values [20]. They concluded that HD <93 has a 90% success rate if calculus size falls in the range of 5-15 mm, but we did not observe any significance of HD values in predicting ESWL outcome.

Park et al. concluded that SSD independently influences the ESWL outcome. Successful cases had a shorter SSD (78.25 ± 12.15 mm) while failed cases had a higher SSD (92.03 ± 14.51 mm) [21]. In our study, SSD had no major impact on the results.

Limitations and recommendations

The present study was conducted on a relatively small population. Increasing the sample size would improve the statistical power of the results. Performing a chemical analysis of the calculi would have added more value to the study. However, due to its unavailability in our institution, it could not be done.

## Conclusions

Nephrolithiasis is one of the most common renal pathologies encountered in routine daily practice, which causes significant morbidity and health burden. Imaging plays a very crucial role in the diagnosis and treatment planning of nephrolithiasis. ESWL is the first choice for treating renal calculi of 2 cm. NCCT-KUB has played a revolutionary role in the evaluation and management of nephrolithiasis. From our study, we have concluded that NCCT-KUB will help in the selection of patients with a favorable prognosis for ESWL, thereby preventing unnecessary procedures and interventions.
